# Annotations

**Published:** 1906-08-11

**Authors:** 


### ANNOTATIONS.

### The Craving for Alcohol.

The pages of a contemporary contain an address by Dr. Harry Campbell upon the craving for alcohol, and also interesting comments upon the address by Sir Samuel Wilks and many others of distinction in the medical profession. Dr. Harry Campbell considers that the desire for alcohol is due largely to its action as a stimulant, while Sir Samuel Wilks and others doubt whether alcohol should be called a stimulant if one intends to convey the idea that its main effect upon the body is that of stimulation. Sir Samuel Wilks believes that it is in the sedative effect of alcohol its main attraction lies. He says that the inmost cry of human nature is with the lotus-eaters, "There is no joy but calm," and quotes the younger Dumas, who considered that "Man drinks because it makes him feel happy. It is not complete felicity, nor absolute forgetfulness, but it is the dulling of thought, the obscuration of consciousness, a mental lethargy." Dr. Robert Hutchison also supports the view that alcohol is used mainly on account of its narcotic action, and points out that in countries where alcohol has been forbidden on religious grounds a powerful narcotic—opium—takes its place. Dr. Hutchison thinks it would be interesting to obtain the opinion of several intelligent drunkards upon the reasons why they are unable to resist over-indulgence in alcohol. Opinions coming from such a source would, no doubt, be interesting, but the analysis of feelings is often difficult. We may be conscious of a sense of well-being or the reverse, but it may be impossible for us to describe the difference between the sensations we experience when we feel in good spirits and those which combine to produce a melancholy mood. The drunkard probably would generally only be able to tell us that alcohol makes him feel happier, and would perhaps rarely attribute with Dumas his happiness to a "dulling of thought" and "mental lethargy." Yet a far older writer than Dumas must have taken this view when of the poor man he said, "Let him drink and forget his poverty and remember his misery no more."

### Cooking in the Army.

The keynote of those who seek to deal with either physical or moral ills is "prevention is better than cure." Surgeon-General Evatt has recently expressed the opinion that much of the drunkenness which occurs in the Army is dependent upon the absence of carefully prepared and well-served meals. He considers that want of efficiency in other directions, such as defective signalling, has killed its tens of soldiers, but that "ignorant, stupid, and dirty cooking has killed its hundreds by driving the men to the canteen after an unsatisfactory meal." Happily there is one school of cookery in the Army, but this is considered to be "beneath notice" and is not mentioned in the Army List. To men of large ideas anything connected with eating may be considered as little better than a necessary evil. We once heard a man describe the dinner-bell, with some measure of sincerity, as a nuisance; but life is made up of little things, and these little things of importance to us all are all important in the eyes of some. There are those who, if they do not find satisfaction in what lies in the routine of their daily life will seek it, probably with harm to themselves, elsewhere. In the interests of the health of the Army the question of providing further means for learning cookery may well be considered. In many of the elementary schools cookery is now taught. This, we may hope, will be an aid to domestic happiness in civil life and that with increased love of home there will be less love for the public-house. What is being done for the civilian may no doubt, with advantage, be extended to his military brother.

### The Preparation of Infants' Foods.

Some mmbers of the medical profession set their face against the use of all patent foods for infants and there is a reason for such an attitude. The painful and dangerous disease of infantile scurvy is rarely seen except in babies fed for some time upon patent foods. This danger is recognised by the manufacturers of infants' foods, and those who use them are recommended to give in addition to the food, articles of diet which are of value in the prevention of scurvy. If these directions are carefully followed, no doubt, in the great majority of instances all risk is removed; yet the fact that the children taking these foods have to be protected from possible danger clearly suggests that the foods should not be continuously used for very many weeks at a time. Yet they undoubtedly are of value. Infants who have failed to improve on cow's milk administered in various dilutions and modifications, often rapidly gain weight when taking a patent food. Recently Messrs Allen and Hanbury gave us, amongst others, an opportunity of inspecting their works situated on the borders of the quiet country town of Ware, and of viewing the preparation of their infants' foods. The cream is first separated from the milk, some of the casein is then removed, and afterwards cream and some malt added. The mixture is then dried. Allenbury's food is, therefore, it is scarcely necessary to remark, a dried modified cow's milk. The drying removes that vital element which seems necessary to prevent the appearance of scurvy, and consequently raw meat-juice or fresh grape-juice are advised also to be given.

